# Hospital Stay of Orthopedic Cases in a Tertiary Care Hospital: A Descriptive Cross-sectional Study

**DOI:** 10.31729/jnma.6400

**Published:** 2021-04-30

**Authors:** Pramod Joshi

**Affiliations:** 1 Department of Orthopedics, Seti Provincial Hospital, Dhangadhi, Nepal

**Keywords:** *hospital stay*, *Nepal*, *orthopedics*

## Abstract

**Introduction:**

Orthopedic conditions includes a range of condition varying from traumatic injuries, congenital anomalies, chronic back pain, arthritis, rheumatologic conditions, and other. Length of hospital stay is determined by a number of factors such as symptom severity, patient co morbidity and hospital availability. Our study aims to study the length of hospital stay of the patients admitted in a provincial hospital.

**Methods:**

A descriptive cross-sectional study was conducted in Seti Provincial Hospital in the month of January among 800 cases. The record of each orthopedic cases admitted in the hospital was retrospectively collected from the medical record section after receiving ethical approval from Institutional Review Committee of Seti Provincial Hospital. Whole sampling technique was used. Data were analyzed by Statistical Package for the Social Sciences version 20. The descriptive statistical analysis was done.

**Results:**

The average length of hospital stay was 2.87 days with the maximum length of the stay of 10 days and the minimum stay of zero days (discharged on the same day). Forearm bone fracture was the main reason for admission in the hospital 325 (40.62%).

**Conclusions:**

Length of the hospital stay was found to shorter than the previous study done in similar settings.

## INTRODUCTION

Orthopedic cases are prevalent and they are the top most cause of long-term sever pain and physical disability and also affect the psychosocial status of the affected people as well as their carer.^1^ Length of Stay is determined by a complex interweaving network of multiple supply and demand factors which operate at macro-, meso-, and micro-levels. Factors Influencing hospital admission and stay includes various factors such as symptom severity, patient co morbidity, bed availability, hospital admission threshold, pre-admission factors, social, economic and cultural, healthcare environment and primary care access factors.^2,3^

Hospitalization is a major risk for older persons, particularly the very old. In many cases, hospitalization is associated with an irreversible decline in functional status, cognitive performance, and quality of life.^4,5^ The study of hospital stay duration and study on clinicodemographic pattern of the orthopedic cases is necessary as it assist in identifying areas for primary prevention.

This study aims to find about the the hospital stay duration of orthopedic cases admitted in the hospital.

## METHODS

A descriptive cross-sectional study was conducted in Seti Provincial Hospital in the month of January. This study included retrospective analysis of the record of admitted orthopedic cases from the last one year. Ethical approval was taken from Ethical Review Board of Nepal Health Research Council (Ref 1604).

All the orthopedic cases admitted in the hospital from the last one year meeting inclusion criteria were included in the study. Record of cases diagnosed with orthopedic conditions was included for the study. However, incomplete Record, record of death cases, duplicate Cases, record with missing diagnoses were excluded from the study.

Sample size calculation,

$$\begin{array}{ll} \text{n} & ={\text{Z}}^{\text{2}}\times \text{σ}/{\text{e}}^{\text{2}}\\ & ={\left(1.96\right)}^{2}\times {\left(0.5\right)}^{2}/{\left(0.04\right)}^{\text{2}}\\ & =600 \end{array}$$

where,

n = minimum sample size
Z = 1.96 at 95 % Confidence Interval
σ = 0.5, standard deviation for maximum sample size
e = margin of error, 4%

However, total sample size taken was 800. The records of admitted orthopedic cases were filled in a specifically designed structured record review form. In order to maintain confidentiality and anonymity, the personal identification details were eliminated, and a consecutive reference number to each case was assigned.

Data was collected in Microsoft Excel and then edited and checked. After that the data was put in SPSS. The descriptive statistical analysis was done. Frequency, percentages were calculated for binary data and mean and standard deviation were calculated for continuous data.

## RESULTS

In this study, we studied the record of eight hundred admitted orthopedic cases in one year duration. The average length of hospital stay came to be 2.87 days in which the maximum length of the stay was 10 days and the minimum stay of the case was zero days (discharged on the same day) (Table 1).

**Table 1:** Summary for length of stay of the hospitalized patients.

S.N	Metric	Value
1.	Mean	2.87
2.	Median	1.5
3.	Minimum	0 (Discharged on the same day)
4.	Maximum	10

Among the cases, male 515 (64.37%) were more than female 285 (35.63%). Most common age group was 15-30 which was 300 (37.5%) followed by 30-45 150 (18.75%), 45-60 140 (17.5%), 0-15 110 (13.75%) and 60+ 100 (12.5%). In terms of address, almost all patients were from Province 7, 718 (89.75%) followed by Province 6 , 56 (7%).

Forearm bone fracture was the main reason for admission in the hospital 325 (40.62%). Humerus Bone Fracture 201 (25.12%) and Tibia and Fibula Fracture 104 (13%) were the second and third most presenting cases. Similarly, road traffic accident 90 (11.25%), cut injury 36 (4.5%), Physical assault 16 (2%), phalanx fracture 14 (1.75%) and fall injury 14 (1.75%) are other common presenting conditions.

In terms of management, almost all cases were managed surgically 756 (94.5%) whereas 44 (5.5%) cases were managed medically.

## DISCUSSION

Orthopedic cases are prevalent and they are the top most cause of long-term severe pain and physical disability and also affect the psychosocial status of the affected people as well as their carer.^1^ Our study finds out about the pattern of hospital stay as well as clinico-demographic pattern of orthopedic admitted cases of a tertiary care centre

Gani, et al. studied pattern and prevalence of orthopaedic cases in a tertiary care centre and found that male patients were more than female with the ratio of 1.45. This findings are similar to our study with the ratio of male to female of 1.80.^6^

A study done from a database of patients in a tertiary general university hospital in South Korea between January and December 2013 showed the average length of hospital stay as 7.0 whereas the median was 4.0.7 The maximum stay was 243 days. In our study, the mean stay was shorter which can be attributed to the fact that our study site is a provincial hospital as compared to the university hospital in the above study. A similar study done in a tertiary care setting of Nepal by Bidary, et al. depicted that the average length of stay in orthopedic cases was 5.0 with minimum of three days and maximum of 10 days.^8^

In a tertiary care centre, Syed, et al. used the 4-year record data to find out that most of the cases presenting comes from age 19-50 years and the most frequent diagnoses belong to lower back pain (25.9%), tendinopathies and enthesopathies (18.3%), bone fractures (11%).^9^ Age group of 15-30 was the commonest in our study whereas the most common reason for admission was forearm bone fracture followed by humerus bone fracture and tibia and fibula bone fracture.

This is a single-centre retrospective study; our study may not be truly representative of the epidemiology and hospital stay of orthopedic problems prevalent in the community. The cross-sectional nature of the study, does not allow the establishment of causality.

## CONCLUSIONS

Length of the hospital stay was found to shorter than research findings in the future, the study involving large area with increased number of sample size is recommended.
